# Bioactive citrate-based polyurethane tissue adhesive for fast sealing and promoted wound healing

**DOI:** 10.1093/rb/rbad101

**Published:** 2023-11-21

**Authors:** Yan Li, Jiawei Liu, Chenxi Lian, He Yang, Mingjiang Zhang, Youfa Wang, Honglian Dai

**Affiliations:** State Key Laboratory of Advanced Technology for Materials Synthesis and Processing, Biomedical Materials and Engineering Research Center of Hubei Province, Wuhan University of Technology, Wuhan 430070, China; State Key Laboratory of Advanced Technology for Materials Synthesis and Processing, Biomedical Materials and Engineering Research Center of Hubei Province, Wuhan University of Technology, Wuhan 430070, China; State Key Laboratory of Advanced Technology for Materials Synthesis and Processing, Biomedical Materials and Engineering Research Center of Hubei Province, Wuhan University of Technology, Wuhan 430070, China; State Key Laboratory of Advanced Technology for Materials Synthesis and Processing, Biomedical Materials and Engineering Research Center of Hubei Province, Wuhan University of Technology, Wuhan 430070, China; State Key Laboratory of Advanced Technology for Materials Synthesis and Processing, Biomedical Materials and Engineering Research Center of Hubei Province, Wuhan University of Technology, Wuhan 430070, China; State Key Laboratory of Advanced Technology for Materials Synthesis and Processing, Biomedical Materials and Engineering Research Center of Hubei Province, Wuhan University of Technology, Wuhan 430070, China; State Key Laboratory of Advanced Technology for Materials Synthesis and Processing, Biomedical Materials and Engineering Research Center of Hubei Province, Wuhan University of Technology, Wuhan 430070, China; Chaozhou Branch of Chemistry and Chemical Engineering Guangdong Laboratory, Chaozhou 521000, China; Shenzhen Research Institute of Wuhan University of Technology, Shenzhen 518000, China

**Keywords:** tissue adhesive, polyurethane, citrate, wet adhesion

## Abstract

As a superior alternative to sutures, tissue adhesives have been developed significantly in recent years. However, existing tissue adhesives struggle to form fast and stable adhesion between tissue interfaces, bond weakly in wet environments and lack bioactivity. In this study, a degradable and bioactive citrate-based polyurethane adhesive is constructed to achieve rapid and strong tissue adhesion. The hydrophobic layer was created with polycaprolactone to overcome the bonding failure between tissue and adhesion layer in wet environments, which can effectively improve the wet bonding strength. This citrate-based polyurethane adhesive provides rapid, non-invasive, liquid-tight and seamless closure of skin incisions, overcoming the limitations of sutures and commercial tissue adhesives. In addition, it exhibits biocompatibility, biodegradability and hemostatic properties. The degradation product citrate could promote the process of angiogenesis and accelerate wound healing. This study provides a novel approach to the development of a fast-adhering wet tissue adhesive and provides a valuable contribution to the development of polyurethane-based tissue adhesives.

## Graphical Abstract

**Figure:**
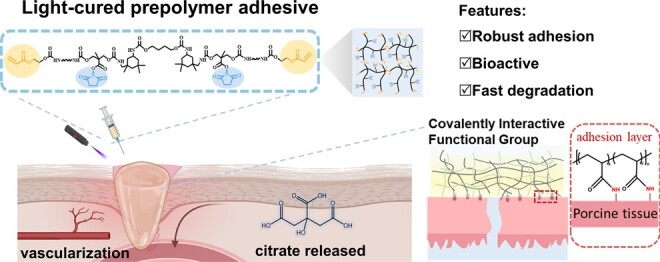

## Introduction

Adhesives are widely used in human life and industrial production. Bioadhesives, such as tissue adhesives, hemostatic agents and tissue sealants, have also made significant strides in recent years. Even though sutures are routinely used to connect damaged tissue and repair surgical trauma, there are still drawbacks, such as overlong operation time, secondary tissue damage and fluid leakage [1, 2]. These challenges make them inadequate for trauma first aid, fragile organs or blood vessels [3]. As the most potential substitute for sutures, tissue adhesive has attracted the attention of many researchers [4–7].

An excellent tissue adhesive for clinical should be biocompatible, degradable and flexible. It also needs strong tissue adhesion property and mechanical strength to accommodate the stretching of the muscles surrounding the injured tissue [7, 8]. Commercial tissue adhesives have been developed with increasing clinical demand for adhesives, and cyanoacrylate glue is the most popular due to its fast curable behavior [9–11]. These adhesives based on the C–C bond are hard degradable, which may inhibit tissue regeneration [8, 12]. Nowadays, most existing advanced tissue adhesives, such as chitosan or gelatin-based hydrogel, dopamine-inspired hydrogel, topo-logical adhesion and other hydrogel bioadhesives are studied for adhesion with various tissues [13–20]. However, slow degradation speed, long bonding and curing time, and uncontrollable oxidation have constrained their application to tissue adhesion [21, 22]. Another challenge for tissue adhesive was the adhesion failure in wet environments. The hydration film formed on the bonded surface reduced the bonding properties and led to bonding failure. Previously, *N*-hydroxysuccinimide (NHS) esters adhesion [23–25] and the addition of hydrophobic components [26–30] were employed to achieve fast bonding and interfacial drainage, which provides a novel strategy for the development of wet adhesives with fast adhesion. However, most of these materials lack biocompatibility and degradability [26, 30]. Thus, it remains a challenge to develop bioactive tissue adhesives with strong tissue adhesion and biodegradability properties.

Degradable polymer-based adhesives have received considerable attention in recent years. These adhesives are mainly based on the main chain of polysaccharides (alginate, chitosan, hyaluronic acid, etc.) or gelatin, which are modified by functionalized pendant groups to achieve adhesion [31, 32]. Compared with synthetic materials, the functionalization and property manipulation of these substances are much more challenging. Among them, the adhesion property is quite difficult to break through. Citrate-based polymers, a class of synthetic bioactive materials [33], have superior biodegradability and biocompatibility and can be functionalized [34–36]. Our previous studies confirmed that citrate-based materials have favorable bioactivity and could promote angiogenesis [37, 38]. Therefore, citrate-based polymers have the potential to serve as a matrix material for tissue adhesives, which can be used to develop bioactive tissue adhesives with superior degradable properties.

**Figure 1.**
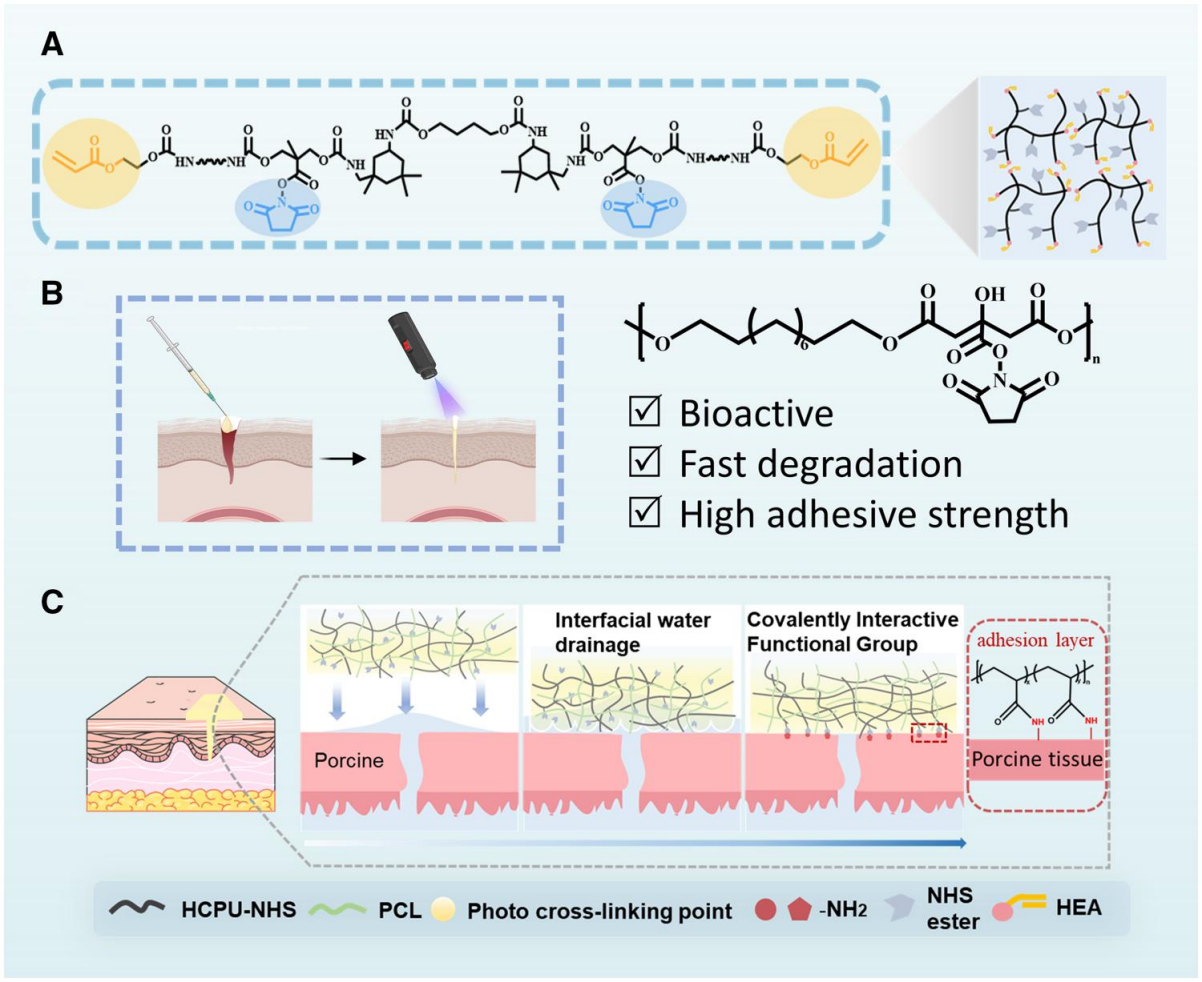
Design of PCL/HCPU-NHS tissue adhesives for would sealing. (**A**) Chemical structure of HCPU-NHS prepolymer. (**B**) By injection of the HCPU-NHS prepolymer and subsequent UV exposure. (**C**) Schematic illustration of PCL/HCPU-NHS tissue adhesives for wound closure by interfacial drainage and covalent cross-linking between the -NHS (polyurethane part) and -NH_2_ (tissue part).

Herein, we developed a fast-degradable citrate-based polyurethane prepolymer adhesive possessing high adhesive strength, biocompatibility and bioactivity. Poly(decamethylene citrate) (PDC), as the soft segment, could improve the bioactivity and degradability of polyurethane simultaneously. Further, the polyurethane adhesive prepolymer was terminated by 2-hydroxyethyl acrylate (HEA) and grafted with NHS esters. The HEA-terminated and NHS esters-grafted polycitrate-based polyurethane (HCPU-NHS) prepolymer exhibited superior injectability, which could be polymerized by photoinitiated free radical. Then, to overcome the influence of the hydration layer on adhesion performance, we introduced polycaprolactone (PCL) into the prepolymer to create a hydrophobic layer for removing the hydration layer on the tissue surface to improve adhesion ability, which increases the contact area between the adhesive and tissue improving adhesion strength (Figure 1). Bioadhesion is achieved through the mechanical interlocking of adhesive prepolymer chains and covalent bonding between -NHS on the adhesive and amine groups (-NH_2_) on tissue surfaces [39]. Besides, the prepolymer chain contains a large number of carbamates, which can interact with skin tissue in the form of hydrogen bonds [6]. Compared to the adhesives using of small molecules, the high viscosity of HCPU-NHS could avoid adhesive leakage and had better biocompatibility. HCPU-NHS has good biocompatibility and brings a significantly lower inflammatory response compared to tissue glue. The degradation product of HCPU-NHS—citrate may contribute to regulating angiogenesis [37, 38]. *In vivo* experiments have demonstrated that these tissue adhesives can be used for hemostasis and wound closure. This PCL/HCPU-NHS adhesive can provide rapid and stable tissue sealing, overcome the disadvantage of traditional polyurethane adhesive, and solve the limitations of suture and commercial tissue adhesives.

## Materials and methods

### Materials

Citric acid (CA), 1,10-decanediol (OD), isophorone diisocyanate (IPDI), 2,2-bis(hydroxymethyl)propionic acid (DMPA), 1,4-butanediol (BDO), 2-hydroxyethyl acrylate (HEA), N-hydroxysuccinimide (NHS) and PCL2000 were bought from Macklin China Corporation. Lithium phenyl (2,4,6-trimethyl-benzoyl) phosphinate (LAP, Z98.0%) and *N*-(3-dimethylaminopropyl)-*N*′-ethylcarbodiimide hydrochloride (EDC·HCL) were obtained from Aladdin Reagent China Company. 3M Vetbond Tissue Adhesive was provided by 3M China. Dulbecco’s modified Eagle’s medium (DMEM) with high glucose was purchased from Gibco. Cell counting kit-8 (CCK-8) and calcein-AM/PI staining kit were purchased from Shanghai Yeasen Biotechnology Co., Ltd. Paraformaldehyde (4%) was purchased from Beijing Solarbio Science & Technology Co., Ltd.

### Preparation of the series of polycitrate-based polyurethane

To prepare the polycitrate-based polyurethane (CPU), 7.8 g polycitrate-decanediol ester (PDC) (780 Da), 2.25 g DMPA, 13.4 ml IPDI was added in 20 ml *N*,*N*-dimethylacetamide (DMAC) in a three-necked flash. The mixture was allowed to stirred at 80°C for 2.5 h under argon. About 1.8 ml BDO was added, and continue the reaction for 2.5 h. HCPU was prepared by adding 1.5 ml HEA at the end of the reaction. Stirring at 70°C for 2 h under argon. The follow-up process is consistent with the above. The prepolymer was obtained after rotary evaporating.

The HCPU-NHS prepolymer was prepared from HCPU prepolymer and NHS. About 25 g prepolymer was dissolved in 40 ml 1,4-dioxane at room temperature. About 4.2 g NHS and 3.5 g EDC·HCL were added into the prepolymer solution, the reaction was performed at 0°C for 2 h, then adjusted the temperature to room temperature for 12 h, and then precipitated in *n*-hexane three times. HCPU-NHS was obtained after rotary evaporation.

About 5 g HCPU-NHS prepolymer and PCL2000 (6–9 wt.%) were dissolved together in 20 ml dichloromethane. The mixed solution was stirred for 2 h at room temperature. The solvent was removed via rotary evaporation. The LAP initiator (0.5 wt.%) was added, and it was stored at 0–4°C in the dark.

### Structure and physicochemical properties characterization

The successful graft and synthesis were analyzed by FT-IR (Thermo Nicolet) (4000–600 cm^−1^ range) and ^1^H NMR (Varian500) spectroscopy (DMSO-d6 as solvent).

The static contact angle test was carried out by the contact angle goniometer (JC2000C 50 Hz). The deionized water droplets are dropped from 10 mm high onto the sample surface. After the droplets were stabilized on the surface, the contact angle was calculated. Each measurement was performed under the same experimental conditions.

The cross-section morphology of HCPU-NHS and PCL/HCPU-NHS (with 6–9 wt.% PCL) films were observed using scanning electron microscopy (SEM, JSM-IT200) with an accelerating voltage of 5 kV.

### Rheological studies

The viscosity of the prepolymer was measured by rotary rheometer (Viscotester IQ) with 25 mm parallel plates (gap 1 mm) by applying shear rates of 0.1–10^3^ S^−1^ at 25°C. To investigate the effects of the blue light irradiation time and the compositions on the rheological properties of polyurethanes at room temperature, a strain-controlled and a 25-mm parallel plate fixture was utilized with time sweep test (gap 1 mm, 25°C, 1 Hz, 1% strain), the real-time changes for storage modulus (*G*′) and loss modulus (*G*″) of the hydrogel irradiated by the blue light (25 mW cm^−2^, 405 nm) were recorded.

### Mechanical tests

Before the tensile test, the polyurethane prepolymer was formed in the mold to make a film of 10 mm × 100 mm and the stress–strain curves of polyurethane were recorded by a universal mechanical testing machine (CMY4503) at a constant deformation rate of 10 mm min^−1^.

### Adhesive ability tests

The samples were measured using fresh porcine skin and were kept moist during the test. The bonding joint between adhesive and procine was made by injecting the adhesive onto fresh procine, and covering it with PVC film, then irradiating it with 405 nm UV light. Lap shear strength was tested according to the ASTM F2255, with 10.0 × 10.0 mm adhesion area and 50 mm/min tensile speed. The shear strength is determined by dividing the maximum force by the adhesion area. Peel strength was tested according to the standard T-peel test ASTM F2256, with 10.0 × 10.0 mm area and 50 mm/min. The maximum interfacial toughness is the maximum peel force divided by the specimen width.

### Swelling ratio

All polyurethane films used were dried to a constant weight. The HCPU-NHS and PCL/HCPU-NHS (with 6–9 wt.% PCL) films (10 mm × 10 mm) were soaked in PBS buffer solution (pH 7.4) and weighed at the pre-set time node. SR = ((Ws − Wd))/Wd × 100%.

### Degradation performance

HCPU-NHS and PCL/HCPU-NHS (with 6–9 wt.% PCL) (10 mm × 10 mm) were soaked in PBS buffer solution (pH = 7.4) at 37°C. At each pre-set node, the films were collected, rinsed with deionized water and dried to a constant weight. Recording their dry weight. Additionally, the degradation liquid of each group was collected, and the pH of the degradation solution liquid was measured using a pH meter. The CA content is tested by CA Colorimetric Assay Kit (E-BC-K351-M).

### Cell compatibility tests

Viability of L929 cells was assessed by the cell counting kit-8 (CCK-8, Yeasen, China) assay. L929 cells were incubated with Roswell Park Memorial Institute 1640 (RPMI-1640, Cytiva, USA) with 10% of fetal bovine serum (FBS, Gbico, USA) in a humidified incubator containing 5% CO_2_ at 37°C. The extract solution was prepared by incubating HCPU-NHS with RPMI-1640 at 37°C for 24 h (0.2 g/ml). For the cell proliferation experiment, L929 cells were seeded in a 96-well plate at a density of 3000 cells/well. The culture medium was replaced by an extract solution after 24 h of culture. After co-incubating for 1, 3 and 5 days, the medium was removed and 20 μl of CCK-8 reagent in 200 μl complete growth medium was then added to each well. After incubating at 37°C for 2 h, 100 μl of the medium in each well was transferred to a 96-well plate (Nest, China). The optical density value (OD value) of each well was calculated at 450 nm with a microplate reader (Thermo Scientific, USA). The control group was the experimental group without materials.

For the cell live/dead experiment, the L929 cells in the well plate on day 3 were stained at 37°C for 15 min with Calcein-AM/PI live/dead cell dye from the LIVE/DEAD^®^ Viability/Cytotoxicity210 Kit and photographed under a fluorescence microscope (Olympus, IX71, Japan).

### *In vitro* angiogenesis study

The human vascular endothelial cells (hUVECs) proliferation assay was similar to the L929 cell assay using F12/DMEM (Gibco, USA) medium with 10% of FBS. The specific steps of the tube-formation assay are as follows: To begin, 100 µl matrix glue was added to each well of a 96-well plate and incubated for 30 min at 37°C. After the matrix glue was cured, 3 × 10^4^ cells (hUVECs) were added to each well. Upon observing the cells adhering, substitute extract solution for DMEM/F12 medium and observe cell morphology under a brightfield microscope after 2 h. The specific steps of the cell-migration assay are as follows: The hUVECs were seeded in 12-well plates at a density of 5 × 10^5^ cells per well. The cells were incubated in a cell incubator (37°C, 5%CO_2_) until the bottom of the well plates had been covered, followed by the central area of the plates was scraped with a 200-μl pipette tip and the well plates were washed with PBS to remove excess cells. In the next step, the initial DMEM medium and leaching solution were added, with the initial DMEM medium also serving as a blank control group. The cell-migration performance was determined by the migration of cells to the blank scratched area by different materials.

### Animal experiments

All the animal experiment protocols were approved by the Institutional Review Committee of the Wuhan University of Technology (WHUT-2022-021). To evaluate the wound healing performance of HCPU-NHS and PCL/HCPU-NHS (with 8 wt.% PCL) adhesives *in vivo*, a full-thickness rat skin incision model was established using female Sprague Dawley rats (SD rats, 240–280 g), and after being paralyzed rats with 5 wt.% chloral hydrate, the rats were fixed on the surgical corkboard and shaved. Four full-thickness incisions (2 cm in length) are created on the back of the rat. It was treated with HCPU-NHS and PCL/HCPU-NHS (with 8 wt.% PCL) adhesives, 3M tissue adhesive and suture group as control. The incisions area was monitored and photographed on days 1, 3, 5, 7, 10, and 12, respectively.

### Histologic and immunohistochemistry

For histological examination, the wound skin tissues were isolated and immediately fixed in 4% paraformaldehyde, and then embedded in paraffin, cross-sectioned to 4 μm thickness slices. They were stained with hematoxylin-eosin and Masson trichrome staining. All the stained histological sections were observed under a microscope.

### Liver injury model

SD rats (200–250 g, females) were used as experimental subjects to evaluate the hemostatic effect of PCL/HCPU-NHS (with 8 wt.% PCL) adhesives. The rats were anesthetized by injection of 5 wt.% chloral hydrate and then fixed on a surgical corkboard. The liver of the rats was exposed through an abdominal incision, and the tissue fluid around the liver was carefully removed. Incisions were made on the liver surface (3 mm in depth and 7 mm in length) resulting in liver bleeding. And immediately covered the bleeding site with a gelatin hemostatic sponge coated with PCL/HCPU-NHS (with 8 wt.% PCL) adhesive. Pre-weighed filter papers were used to absorb blood around the wound, the filter papers were weighed, and the control group was a gelatin hemostatic sponge not coated with PCL/HCPU-NHS (with 8 wt.% PCL) adhesive.

### Statistical analysis

All the data were processed with Origin 2021 and presented as the mean ± standard deviation. One-way analysis of variance (one-way ANOVA) was used to determine the significance level between multiple groups, and the significance level was considered as *P < 0.05, **P < 0.01, ***P < 0.001.

## Results and discussion

### Design and characteristics of HCPU-NHS

**Figure 2.**
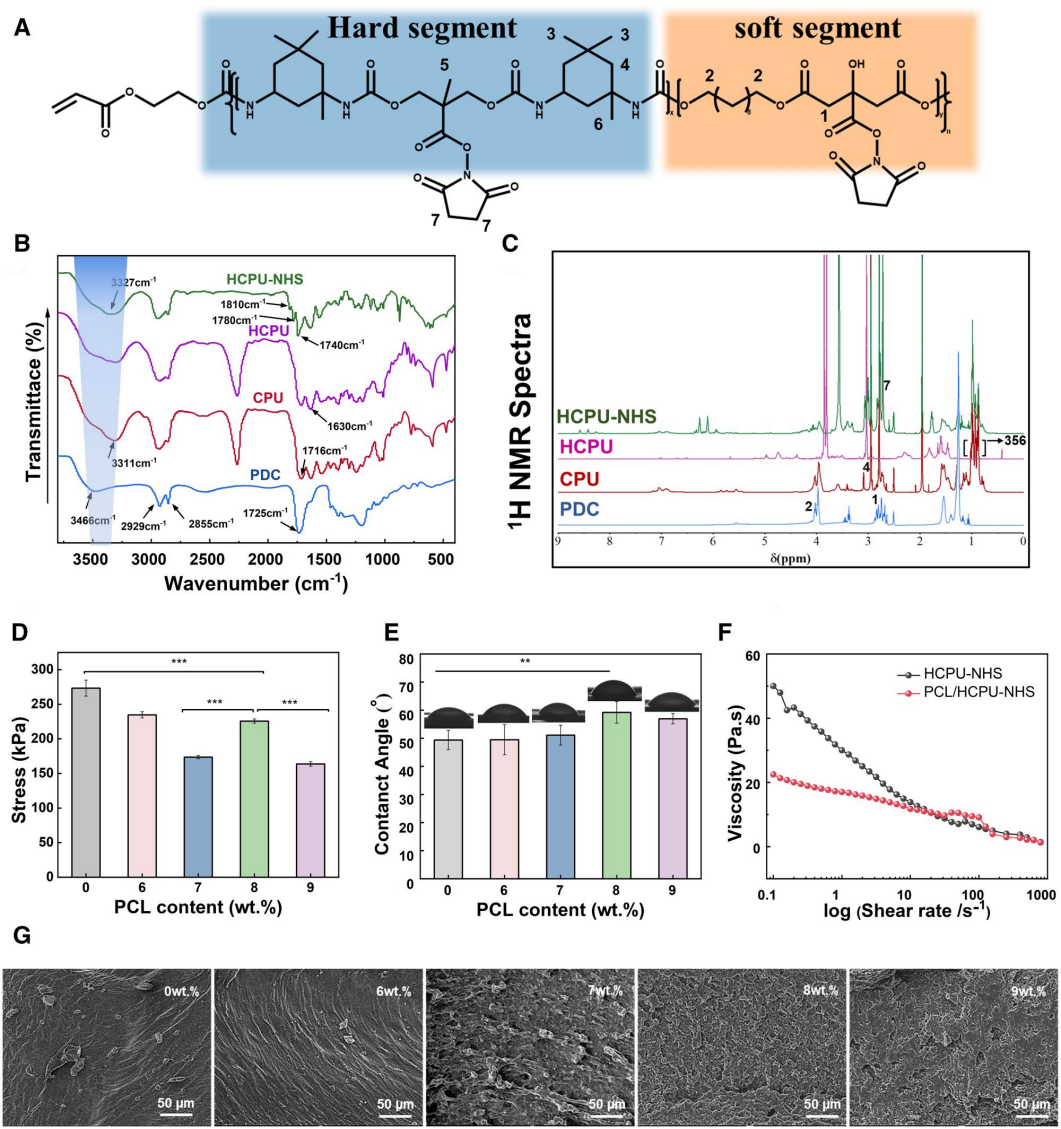
The structure and physicochemical properties of HCPU-NHS and PCL/HCPU-NHS. (**A**) Chemical structure of HCPU-NHS, where *x* is the repeating number of hard segments, *y* is the repeating number of soft segments and *n* is the repeating number of the PU segments. (**B**) The FT-IR spectra of PDC, CPU, HCPU and HCPU-NHS. (**C**) 1H NMR spectrum of PDC, CPU, HCPU and HCPU-NHS. (**D**) The tensile strength of HCPU-NHS and PCL/HCPU-NHS (with 6–9 wt.% PCL). (**E**) The water contact angle of HCPU-NHS and PCL/HCPU-NHS (with 6–9 wt.% PCL). (**F**) The viscosity of HCPU-NHS and PCL/HCPU-NHS. (**G**) SEM images (from right to left) of the fractured surfaces of HCPU-NHS and PCL/HCPU-NHS (with 6–9 wt.% PCL).

The citrate-based polyurethane prepolymer adhesive tissue was based on the degradability polymer PDC as soft segment and terminated by HEA and grafted with NHS esters to achieve photo-crosslinking and adhesion. To verify the successful preparation of CPU, HCPU and HCPU-NHS, the structures of polymers were investigated using FT-IR and 1H NMR (Figure 2B and C). The FT-IR and ^1^H NMR spectra of the PDC are consistent with existing studies [40]. The FT-IR spectrum of CPU exhibited vital spectral absorbance features at 3311 cm^−1^ and 1716 cm^−1^, which respectively assigned to N–H stretching vibration of aliphatic secondary amine and C = O stretching vibration of the esters and carboxyl [53]. The characteristic peak at 1630 cm^−1^ was attributed to the C = C stretching vibration and the characteristic peak at 1810, 1780 , and 1740 cm^−1^ was attributed to the NHS esters of HCPU-NHS [41]. Further, the ^1^H NMR showed the chemical shift at 2.75 ppm was attributed to the -NHS esters of HCPU-NHS, indicating that NHS was successfully grafted onto HCPU. The peak at 2.95 ppm was assigned to the CH_2_ group in IPDI and PDC. Then, the chemical shift value of about 0.98 ppm belongs to the -CH_3_ in the structure of IPDI and DMPA [53] (Figure 2C). The FT-IR and ^1^H NMR demonstrated the successful synthesis of HCPU prepolymer.

According to the previous study, the hydration layer between the subtract and adhesives is one of the main factors affecting the adhesion performance [26]. To overcome the influence of the hydration layer, hydrophobic polymer PCL was introduced to the polyurethane. Firstly, the effect of PCL on the mechanical properties of the adhesive was studied. With the increasing content of PCL, the tensile strength of the selected components showed a trend of first decreasing, then increasing and then decreasing. The tensile strengths of HCPU-NHS and 6–9 wt.% PCL/HCPU-NHS were 273.33 ± 11.59, 234.67 ± 4.50, 173.67 ± 2.30, 225.67 ± 3.05, and 163.67 ± 3.21 kPa, respectively (Figure 2D). However, the elongation at break increased with the increase of blending content (Supplementary Figure S2A). PCL has a toughening ability, which can enhance the elongation at break of the material, but may weaken the tensile strength to some extent [42, 43]. In general, after adding PCL, the tensile strength of each group was lower than that of pure HCPU-NHS, and the optimal content was 8 wt.%, which showed the highest tensile strength. So, we could consider it the group with the best physical blend compatibility among the high blends of the selected components [44].

In order to further explore the mechanism of PCL on adhesion, the water contact angle of each component was analyzed. The HCPU-NHS and 6–9 wt.% PCL/HCPU-NHS were 49.33 ± 3.44°, 49.43 ± 5.37°, 51.51 ± 3.56°, 59.2 ± 3.87°, 56.93 ± 1.82°, respectively (Figure 2E). The water contact angle of 8 wt.% PCL/HCPU-NHS is the largest and has increased by 10 degrees compared to HCPU-NHS. It confirms that adding hydrophobic polymer PCL improves the hydrophobicity. The hydrophobicity of solid surfaces depends on their inherent characteristics, surface smoothness [44, 45]. Whether the surface composition of the solid is uniform and regularly distributed will have a substantial impact on its hydrophobicity. The SEM image showed that 8 wt.% PCL/HCPU-NHS possessed a uniform structure (Figure 2G). Combined with the SEM images and tensile strength, the 8 wt.% PCL/HCPU-NHS is the most compatible of the high blends and has the most homogeneous PCL distribution. It is also worth noting that the addition of PCL reduces the viscosity of the adhesive (Figure 2F), which allows the adhesive to better penetrate the tissue surface and strengthen the bond between the polymer and the matrix.

The swelling properties of materials are important for tissue adhesives, so we investigated the swelling properties of PCL/HCPU-NHS with different blending content. The swelling rate of HCPU-NHS and 6–9 wt.% PCL/HCPU-NHS were 245.30 ± 7.50, 130.53 ± 16.38, 141.20 ± 20.36, 146.5 ± 9.63, 145.87 ± 7.10%, respectively (Supplementary Figure S2B). The addition of PCL also reduces the swelling rate of the adhesive due to its hydrophobic effect. The low swelling of adhesive could reduce the weakening of adhesion strength and prevent the rupture and re-bleeding of the wound.

To study the injectability and curing speed of the prepolymer, the rheological behavior was studied. Figure 2F shows the viscosity of the prepolymer. Adding PCL decreased the viscosity in the low shear rate range (0.1–10 S^−1^). With the increase of shear rate, the viscosity decreases, which indicates that the prepolymer has the characteristic of shear thinning. It is demonstrated that the prepolymers with and without PCL are both extrudable, which can be filled into irregular wounds (Supplementary Figure S1). The adhesive could be crosslinked under 405 nm LED light. According to the rheological curves, the modules of the prepolymer increased immediately after the irradiation (Supplementary Figure S2D). It is worth noting that the intensity of the UV light used in the rheological test was lower, so the elastic modulus reaches its maximum at around 4 min. The curing time of the prepolymer adhesive in other tests is lower than the measured value in the rheology.

### Tissue adhesive properties

**Figure 3.**
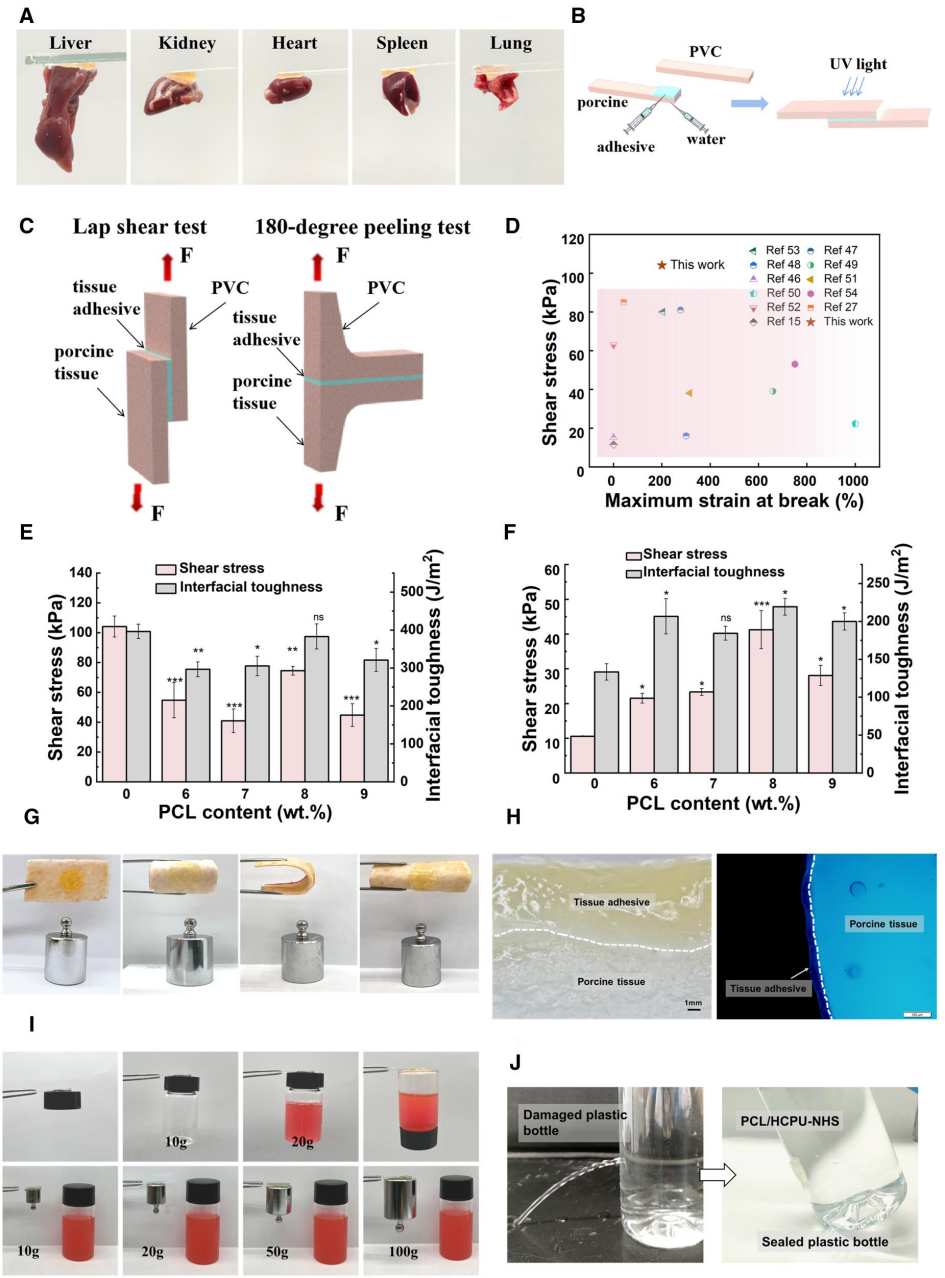
Adhesion performance of the PCL/HCPU-NHS tissue adhesives. (**A**) Adhesive properties to various tissues. (**B**) Schematic diagram of adhesive adhesion. (**C**) Schematic illustration of the lap shear and the 180° peeling tests used here for assessing the shear strength and interfacial toughness, respectively. (**D**) Comprehensive comparison between HCPU-NHS and the reported tissue adhesive regarding the adhesion strength and fracture strain. (**E**) The shear strength and interfacial toughness of the HCPU-NHS and PCL/HCPU-NHS (with 6–9 wt.% PCL) adhesive. (**F**) The shear strength and interfacial toughness of the HCPU-NHS and PCL/HCPU-NHS (with 6–9 wt.% PCL) adhesive in wet. (**G**) The HCPU-NHS adhesive adheres to the surface of the porcine and bends. (**H**) Photographs of the adhesion interface between the HCPU-NHS (with 8 wt.% PCL) adhesive and porcine tissues. (**I**) The adhesion of HCPU-NHS adhesives to plastics and metals. (**J**) The PCL/HCPU-NHS (with 8 wt.% PCL) can effectively seal a leaky plastic bottle filled with water.

The HCPU-NHS tissue adhesives could produce rapid tissue adhesion in various soft tissues such as heart, liver, spleen, lung and kidney (Figure 3A). To evaluate the adhesion properties between tissues, the adhesive was injected onto the surface of porcine, covered with PVC sheet and then exposed to UV light (Figure 3B). The adhesive strengths of HCPU-NHS and PCL/HCPU-NHS (with 6–9 wt.% PCL) adhesives were measured by lap shear and peeling tests (Figure 3C and Supplementary Figure S3). The wet adhesion of the adhesive was evaluated by adding 3–5 drops of deionized water to the surface of fresh and wet porcine to create a wet environment, based on which the shear strength and peel strength was tested [13].

The dry shear strength of HCPU-NHS and 6–9 wt.% PCL/HCPU-NHS were 104.13 ± 7.04, 54.80 ± 11.78, 41.00 ± 7.94, 74.55 ± 2.95, 44.80 ± 7.61 kPa, respectively (Figure 3E). The decreasing of adhesive strength is because of the nonadhesive property of PCL. Compared to the adhesive previously reported, our adhesive lap shear strength was higher than most previous tissue adhesives (Figure 3D) [15, 27, 46–54]. The wet shear strengths of HCPU-NHS and 6–9 wt.% PCL/HCPU-NHS were 10.57 ± 0.15, 21.53 ± 1.40, 23.33 ± 1.00, 41.25 ± 6.85, 28.07 ± 2.90 kPa, and the wet interfacial toughness of HCPU-NHS and 6–9 wt.% PCL/HCPU-NHS were 133.33 ± 10.79, 206.67 ± 23.18, 184.33 ± 9.07, 219.33 ± 11.02, 200.00 ± 11.35 J/m^2^, respectively (Figure 3F and Supplementary Figure S4). The results demonstrated that after adding the hydrophobic polymer PCL, the wet adhesion of each group was higher than that of the HCPU-NHS adhesive. The addition of 8 wt.% PCL showed the highest wet adhesion strength. The adhesion performance of these tissue adhesives is determined by their own cohesion and covalent bonding strength with the tissue surface, besides the wet adhesion strength is affected by the hydrophobicity of the adhesive [55]. The PCL/HCPU-NHS (with 8 wt.% PCL) had the larger tensile strength and the largest water contact angle, showing higher cohesion and hydrophobicity in the study group. Besides, interfacial toughness results showed no significant difference between 6 and 9 wt.% groups, and the difference was lower than shear strength. The interfacial toughness obtained by the peel test is less related to the cohesion strength of the material than shear strength, is more related to the adhesion strength between the adhesive and the tissue interface [6]. The above results demonstrated that the addition of PCL can increase hydrophobicity and improve adhesion strength. The PU adhesive adhered to the porcine skin, which remained attached without detachment after stretching, bending and distorting (Figure 3G). Light microscopy and fluorescence microscopy images further demonstrate the conformity of the adhesion interface between adhesive and porcine tissue (Figure 3H). Additionally, owing to the hydrogen bonding provided by the carbamate, the citrate-based polyurethane tissue adhesive was capable of creating adhesive joints between biological tissue and common engineering solids, including glass, plastic, metal, etc., and could bear 100 g weight without bond failure and detachment (Figure 3I). We then adhered a PVC film with a layer of PCL/HCPU-NHS (with 8 wt.%PCL) adhesive onto a leaking plastic bottle (diameter of the hole was 5 mm) to successfully seal the leakage (Figure 3J).

### Biodegradability and cytocompatibility

**Figure 4.**
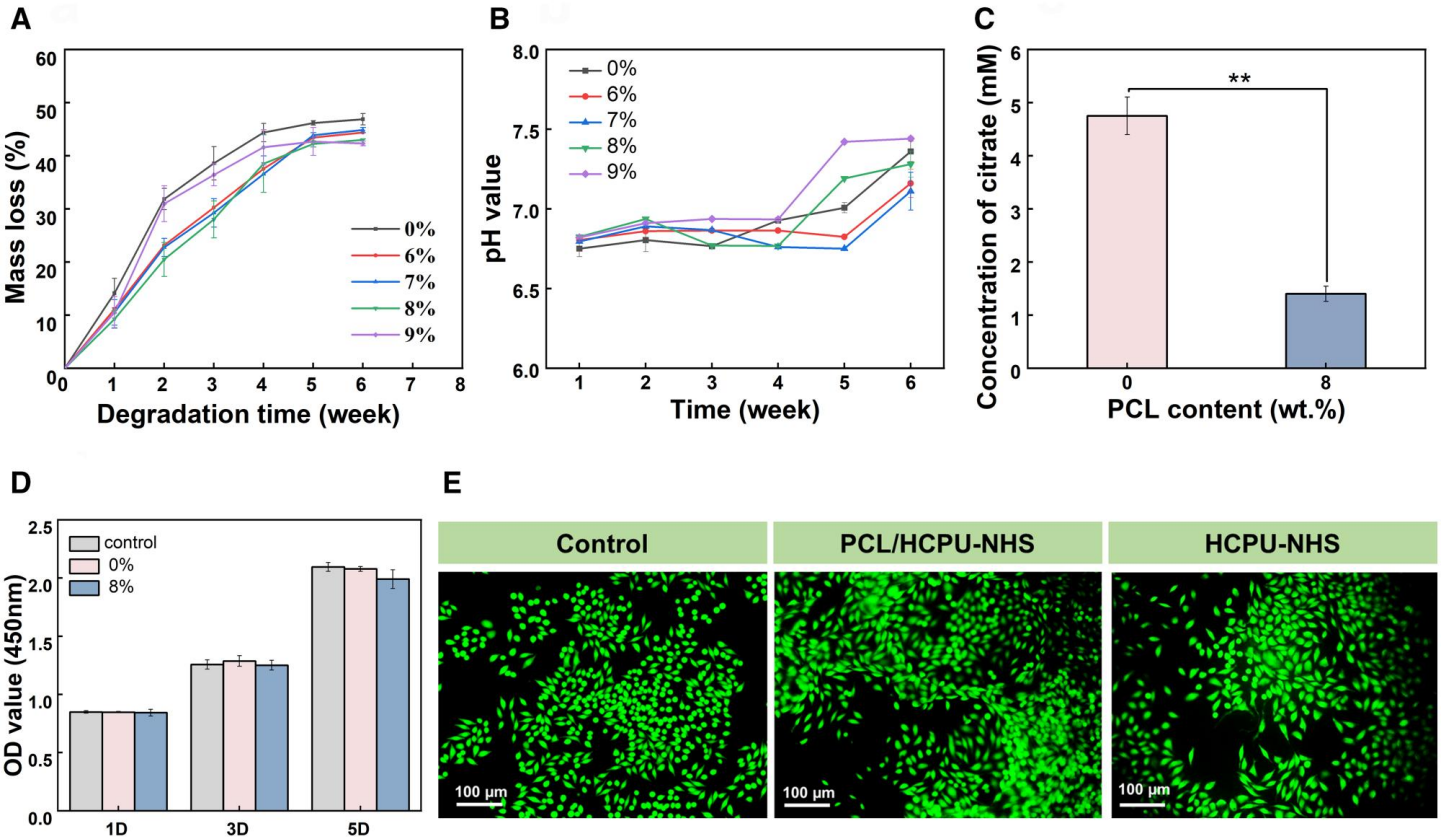
The biodegradation and cytocompatibility of HCPU-NHS and PCL/HCPU-NHS (with 6–9 wt.% PCL). (**A**) The mass loss curves of the HCPU-NHS and PCL/HCPU-NHS(with 6–9 wt.% PCL). (**B**) The pH value of degradation liquid. (**C**) The citrate content of degradation liquid of HCPU-NHS and PCL/HCPU-NHS (with 8 wt.% PCL). (**D**) The OD value of L929 cells was obtained by leaching solution method for 1, 3 and 5 days. (**E**) Live/dead staining of L929 cells after co-culture with material for 3 days.

Traditionally, wound closure requires additional post-healing procedures to remove wound closure devices and debris after healing, the design of an adhesive with biodegradable behavior was highly desired. To simulate the degradation environment *in vivo*, we investigated the hydrolytic degradation properties of HCPU-NHS and PCL/HCPU-NHS (with 6–9 wt.% PCL) in PBS. Within 6 weeks, the weight loss rate of all components can reach 40% or more (Figure 4A). The degradation rate of HCPU-NHS and 6–9 wt.% PCL/HCPU-NHS were 46.86 ± 1.07, 44.43 ± 0.55, 44.82 ± 0.50, 42.96 ± 0.23, 42.28 ± 0.44%, respectively. The degradation rate of PCL/HCPU-NHS (with 6–9 wt.% PCL) adhesive was lower than that of pure polyurethane tissue adhesive. Consequently, the degradation rate of HCPU-NHS is faster than PCL2000, and much faster than most existing polyurethane biomedical materials [56, 57]. We also found that the degradation rate of 8 wt.% PCL/HCPU was the slowest in the first 2 weeks. The degradation behavior of polymers may depend on the hydrophilicity of their surfaces. Due to its higher hydrophobicity, the upfront degradation rate is lower, but after further degradation, the surface morphology was changed, and the rate of weight loss began to increase after the third week.

In addition to mass loss, the pH of the degradation liquid is also crucial. Citrate is a key component of degradation products, and its acidity may affect the application of adhesives in tissue adhesion and wound healing. We collected the degradation liquid of all the components and measured their pH values. From Figure 4B, the pH value at each time point was between 7 and 6.7 within 4 weeks, and the closed wound environment was mildly acidic (6.4 ± 0.5), which could provide an ideal wound healing environment [58]. At the later stage of degradation, the soft segment of PDC gradually degraded, resulting in a pH value that gradually approaches 7.4 (pH of PBS buffer solution). The pH value of each time period of all components can be related to the degradation rate of the time period. Thus, the faster the degradation rate, the lower the pH of the degradation solution.

In order to complete the wound healing process, the PU tissue adhesive should be biocompatible and permit the cells in the injured area to migrate and proliferate. Thus, we co-cultured the L929 cells with pure HCPU-NHS and PCL/HCPU-NHS (with 8 wt.%PCL) adhesive samples extract to evaluate *in vitro* biocompatibility and cell behaviors. The OD values of the pure HCPU-NHS and PCL/HCPU-NHS (with 8 wt.%PCL) group were not significantly different from the control group (Figure 4D), indicating that the materials are not cytotoxic. We observed a uniform disperse of L929 cells on the sample substrate, exhibiting normal cell morphologies. The L929 cells in all samples appeared spindle -shaped, with high density and had few dead cells (Figure 4E). There were no significant differences in cell compatibility between the two groups.

### Ability to promote angiogenesis

**Figure 5.**
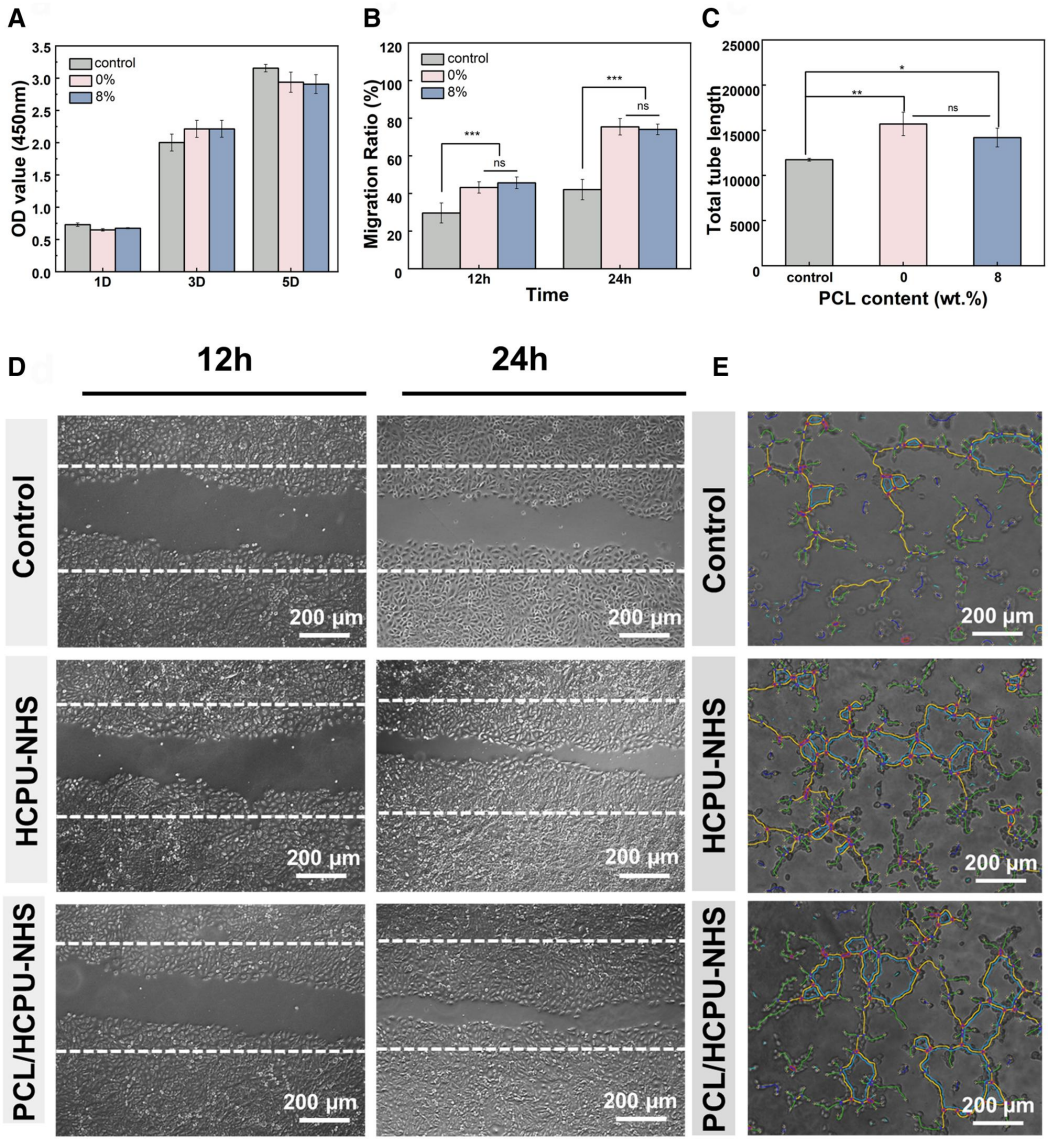
*In vitro* angiogenesis effect of HCPU-NHS and PCL/HCPU-NHS (with 8 wt.% PCL). (**A**) CCK-8 assay of HUVECs proliferation. (**B**) Ratio of the cell-migration area to scratch area. (**C**) Quantitative analysis of total tube length in different group. (**D**) Cell-migration fluorescence images of HUVEC cells for 12, 24 h. (**E**) Images of the tube-formation assay with the different group.

Previous studies have confirmed the important role of citrate in promoting vascular regeneration [35, 37]. The vascular endothelial cell proliferation, migration and tube formation assays were used to evaluate the performance of PCL/HCPU-NHS. According to the results of the cell proliferation experiments, HCPU-NHS and PCL/HCPU-NHS (with 8 wt.% PCL) have no significant cytotoxicity (Figure 5A). They could promote the migration of vascular endothelial cells. The migration ratio of HCPU-NHS and PCL/HCPU-NHS (with 8 wt.% PCL) were 75.38 ± 4.37% and 74.00 ± 2.84%, respectively, which were significantly higher than those in the control group at 42.03 ± 5.44% (Figure 5B and D). Meanwhile, both adhesives could promote the formation of hUVECs into a tubular morphology (Figure 5C and E). Moreover, tube formation assays demonstrated that the total length of formed tubes was more superior to those of the control group (Figure 5C). According to the results, there were no significant differences between the two groups. Previous studies have reported that citrate enhances angiogenesis-related gene expression such as VEGF, TGF-β and bFG in the 0.2–5 mM range [37, 38]. After testing the concentration of citrate in the degradation liquid of both groups in the first week, the concentrations of citrate in the degradation liquid of the HCPU-NHS and PCL/HCPU-NHS (with 8 wt.%PCL) groups were 4.8 ± 0.35 mM and 1.4 ± 0.14 mM, respectively, which was suitable for angiogenesis (Figure 4C). Overall, the HCPU-NHS and PCL/HCPU-NHS (with 8 wt.%PCL) are propitious to angiogenesis, with good bioactivity.

### Wound healing and hemostasis study

**Figure 6.**
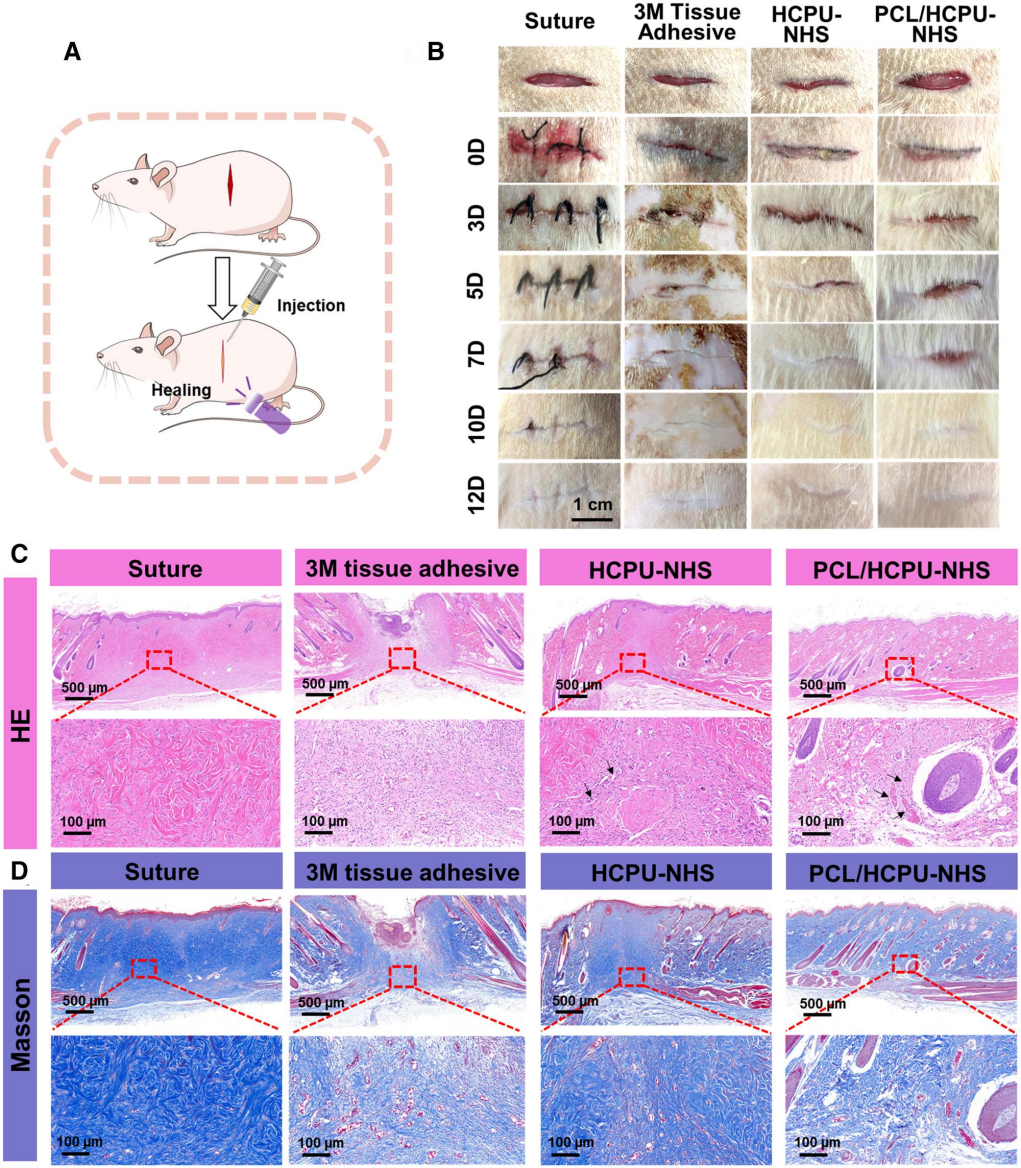
Wound closure assessment of skin incision. (**A**) Schematic diagram of rat wound closure model. (**B**) Representative images of the skin incisions treated by surgical sutures, 3 M, HCPU-NHS, PCL/HCPU-NHS (with 8 wt.%) tissue adhesive. (**C**) H&E staining images and (**D**) Masson’s trichrome staining of the skin incisions on the 12th day.

Moreover, HCPU-NHS and PCL/HCPU-NHS (with 8 wt.%PCL) adhesives were evaluated for the performance of tissue adhesion and wound closure in a full-thickness skin incision model. An incision 2 cm long was made on the back of SD rats. The incisions were closed with HCPU-NHS and PCL/HCPU-NHS (with 8 wt.%PCL) adhesive, in which surgical sutures and 3 M tissue adhesive were negative and positive controls, respectively. A stable bond could achieved, after applying the adhesive to the wound incision and exposed under 405 nm UV irradiation for 30 s to achieve a stable bond, which is very fast and convenient (Figure 6A). As illustrated in Figure 6B, by repairing the incision with 3M tissue adhesive, the surrounding skin which adheres to the adhesive becomes very hard and falls off, owing to the high liquidity of 3M tissue glue. Besides, the 3M tissue glue is a kind of cyanoacrylate adhesive, during the process of wound closure, there is an exothermic reaction that raises the temperature around the skin. Sutures may also damage the skin at the edge of the wound, resulting in secondary tissue damage and the operation time is long and the process is complex. After 10 days, the incisions treated with HCPU-NHS or PCL/HCPU-NHS (with 8 wt.%PCL) adhesives were almost completely closed. In contrast, the wounds in the suture and 3 M tissue adhesive group had not been entirely closed. In the HCPU-NHS, PCL/HCPU-NHS (with 8 wt.%PCL) adhesives and suture group, the wounds healed completely after 12 days, but the scars in the 3M adhesives group were obvious.

Histological analysis was performed on day 12 to further evaluate the wound healing performance, as shown in Figure 6C, H&E staining indicated that the 3M adhesive group had not healed completely, and there was an obvious scar, and even some glue fragments remained [14, 59]. In the suture group, though no obvious inflammation was observed, poor tissue reconstruction was observed. There was no significant recovery of the follicles, which is similar to the previous study [15]. In contrast, the HCPU-NHS or PCL/HCPU-NHS (with 8 wt.% PCL) adhesives exhibited no significant inflammation responses with highly integrated epithelium and more hair follicles than the control group. Remarkably, the incisions treated with PCL/HCPU-NHS (with 8 wt.% PCL) recovered to the same as normal tissue [60]. From the two groups of adhesives, the PCL/HCPU-NHS (with 8 wt.% PCL) adhesive all had obvious angiogenesis, which was consistent with the *in vitro* angiogenesis assay results. Additionally, Masson’s trichrome staining for collagen was performed to evaluate tissue reconstruction. As shown in Figure 5D, the 3 M adhesive and surgical suture groups showed poor tissue reconstruction after 12 days, while the HCPU-NHS or PCL/HCPU-NHS (with 8 wt.% PCL) displayed more collagen deposition [61–64]. The synthetic adhesive exhibited good biocompatibility and promoted angiogenesis and hair follicle regeneration, and these results were consistent with *in vitro* results.

**Figure 7.**
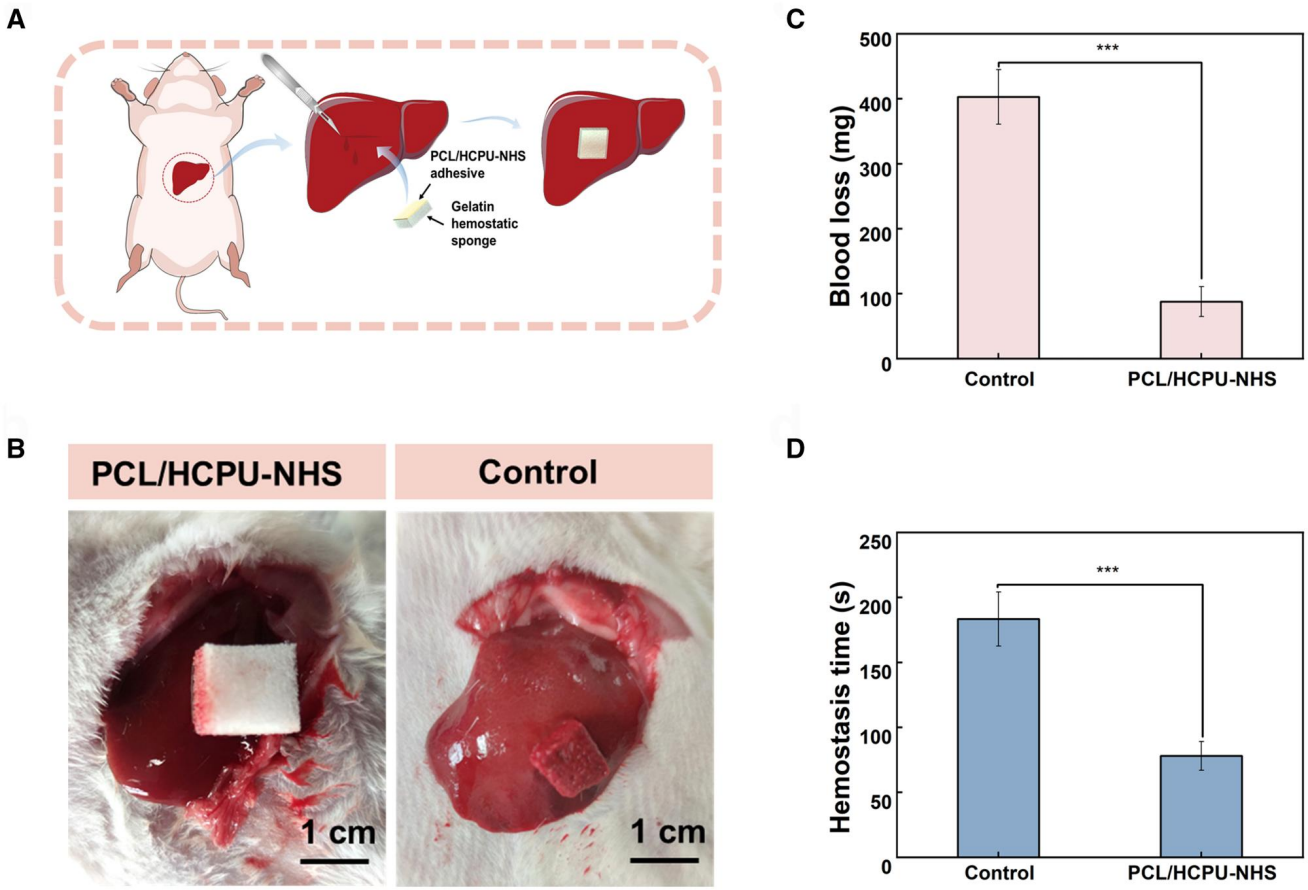
*In vivo* hemostatic capacity evaluation. (**A**) Schematic illustration of the hemostasis by using the gelatin sponge coated with PCL/HCPU-NHS (with 8 wt.%) tissue adhesive in rat liver hemorrhage model. (**B**) Rat liver hemorrhage model. (**C**) and (**D**) Blood loss and hemostatic time in the rat liver hemorrhage model (*n* = 3).

The hemostasis performance of the material *in vivo* was evaluated in the rat hemorrhaging liver model. The PCL/HCPU-NHS (with 8 wt.%PCL) adhesive was coated on a hemostatic gelatin sponge, and an uncoated gelatin sponge was used as a negative control (Figure 7A) [18]. By adhering firmly to the liver surface of the hemorrhage site, the adhesive can create a physical barrier to concentrate blood coagulation factors and collect platelets/erythrocytes to speed up hemostasis that can speed up hemostasis [12, 53, 65]. We made a 7-mm-long and 3-mm-deep incision on the surface of the rat liver, leading to immediate bleeding (Figure 7B). The hemostasis time of the gelatin sponge without PCL/HCPU-NHS (with 8 wt.% PCL) adhesive was 183 s, while the gelatin sponge coated with PCL/HCPU-NHS (with 8 wt.% PCL) adhesive was able to stop bleeding within 78 s (Figure 7D). Compared with the control group, blood loss was decreased (87 and 403 mg) (Figure 7C). All this may demonstrate that adhesives display excellent hemostasis.

## Conclusion

To conclude, we have synthesized a citrate-based photocurable polyurethane tissue adhesive, which possesses favorable biocompatibility, biodegradability, bioactivity and strong adhesive properties, and can be applied to hemostasis and wound closure. The successful introduction of the NHS active ester into the polyurethane chain and blended with the hydrophobic polymer PCL provided stable adhesion to wet tissues. *In vitro* and *in vivo* experiments have confirmed that polyurethane adhesives could strongly adhere to the surface of dry and wet tissue, accelerate wound healing, promote angiogenesis and provide effective hemostatic properties. The effect is significantly superior to the existing commercial adhesive and hemostatic sponge. In this way, we provide valuable development for the design of polyurethane-based tissue adhesives. In addition, our polyurethane adhesive can complete half of the degradation within approximately 6 weeks after implantation. By adjusting the blended or soft segment polymer, *in vivo* biodegradation kinetics can be modulated to improve repair efficacy.

## Supplementary Material

rbad101_Supplementary_Data
