# Supplementary material for: Bioactive citrate-based polyurethane tissue adhesive for fast sealing and promoted wound healing
Source: Regen Biomater. 2023 Nov 21;11:rbad101. doi: 10.1093/rb/rbad101 (PMC10761209; doi:10.1093/rb/rbad101)
Supplement: rbad101_Supplementary_Data [file rbad101_supplementary_data.docx]

Supporting Information:

**Bioactive Citrate-based Polyurethane Tissue Adhesive for Fast Sealing and Promoted Wound Healing**

Yan Li ^‡, a^, Jiawei Liu ^‡, a^, Chenxi Lian ^a^, He Yang ^a^, Mingjiang Zhang ^a^, Youfa Wang ^a^, Honglian Dai ^a ,b, c,*^

^a^ State Key Laboratory of Advanced Technology for Materials Synthesis and Processing, Biomedical Materials and Engineering Research Center of Hubei Province, Wuhan University of Technology, Wuhan 430070, China

^b^ Chaozhou Branch of Chemistry and Chemical Engineering Guangdong Laboratory, Chaozhou 521000, China

^c^ Shenzhen Research Institute of Wuhan University of Technology, Shenzhen 518000, China

‡ Y. L and J. L contributed equally to this work.

**Keywords：** tissue adhesive, polyurethane, citrate, wet adhesion

The PDF file includes

Figure S1 to S4


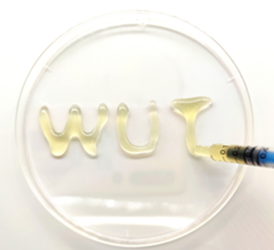


**
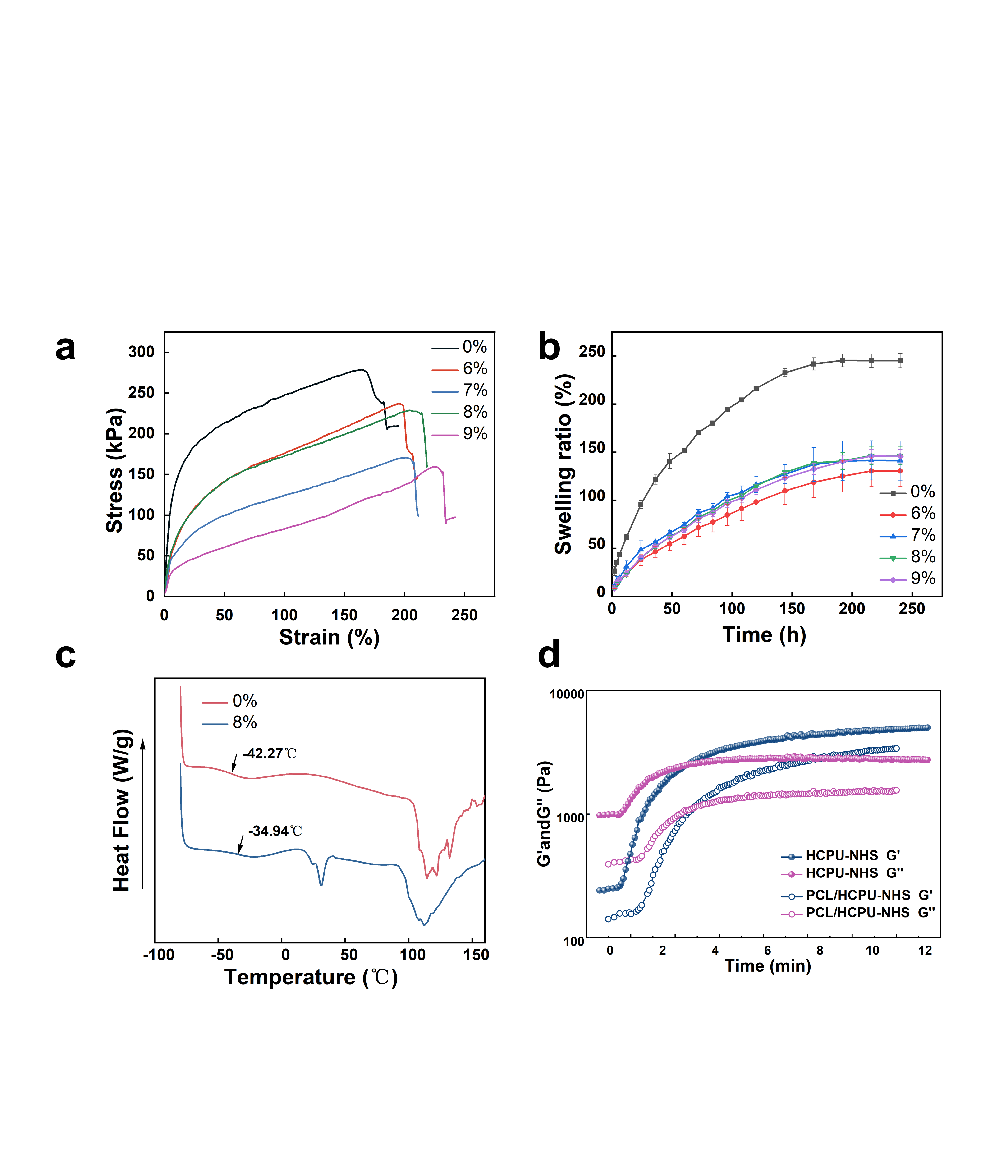
Figure S1.** Injectable ability of HCPU-NHS

**Figure S2.** a) Typical stress–strain curves of HCPU-NHS and PCL/HCPU-NHS (with 6-9 wt.% PCL). b) Swelling ratio (SR) of HCPU-NHS and PCL/HCPU-NHS (with 6-9 wt.% PCL). c) The differential scanning calorimetry of HCPU-NHS and PCL/HCPU-NHS (with 8 wt.% PCL). d) Rheological property of the HCPU-NHS and PCL/HCPU-NHS (with 8 wt.% PCL) under irradiation of 405 nm UV light .


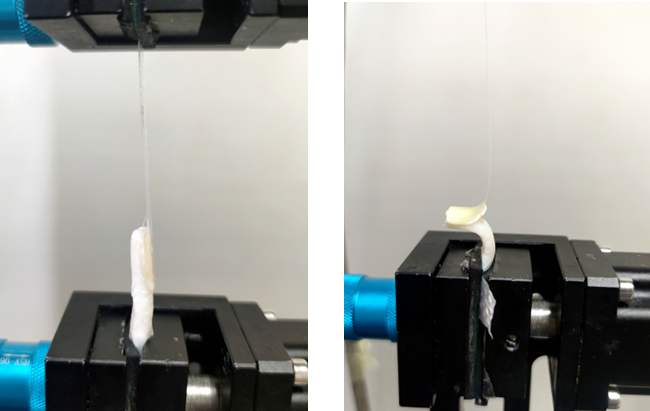


**Figure S3.** lap shear(left) and the 180° peeling tests(right).


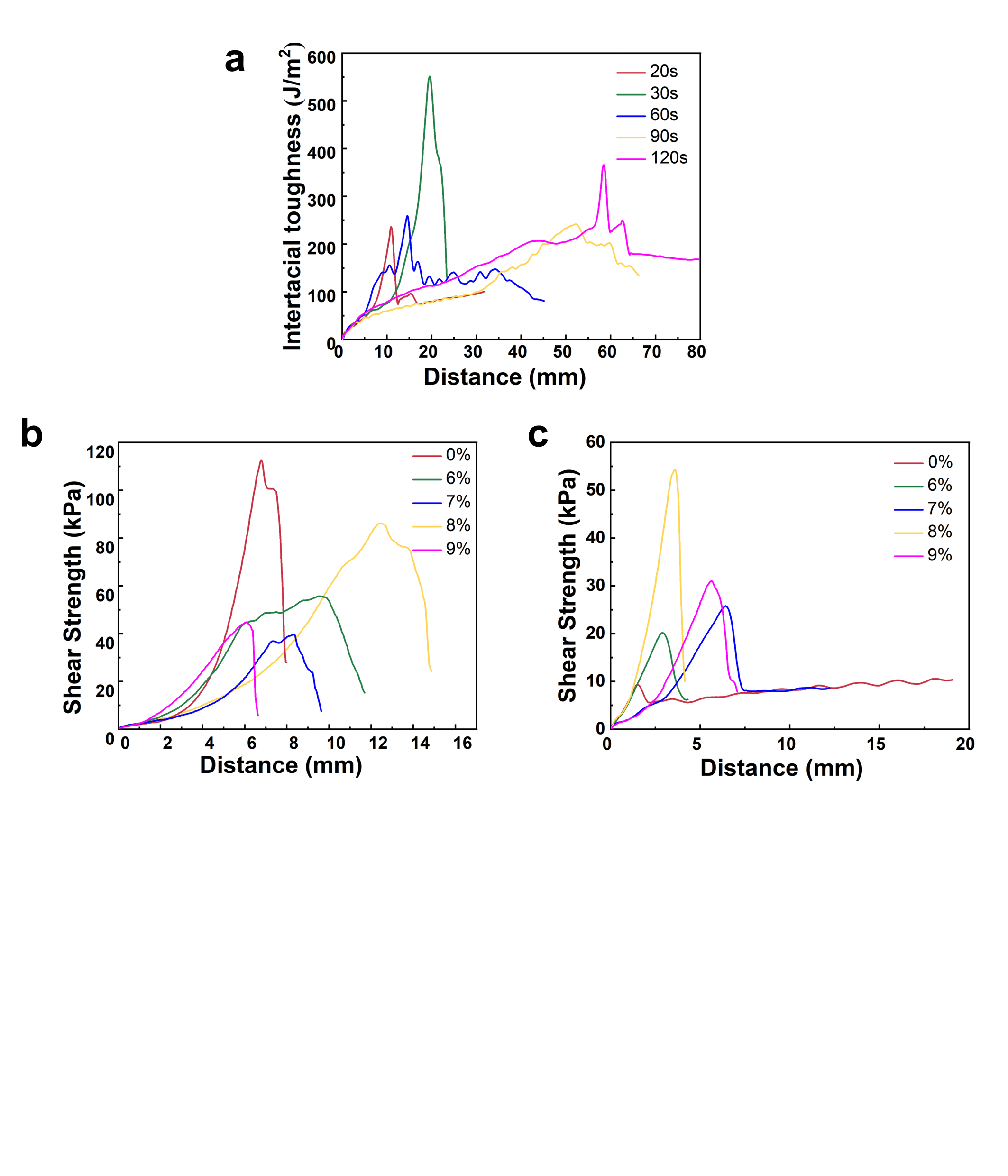


**Figure S4.** a) The interfacial toughness curve of HCPU-NHS adhesion to porcine skin under different light conditions. b) The shear strength curve of HCPU-NHS and PCL/HCPU-NHS (with 6-9 wt.% PCL) adhesion to porcine skin. c) The shear strength curve of HCPU-NHS and PCL/HCPU-NHS (with 6-9 wt.% PCL) adhesion to porcine skin in wet.
